# Supplementary material for: 3-D vascularized breast cancer model to study the role of osteoblast in formation of a pre-metastatic niche
Source: Sci Rep. 2021 Nov 9;11:21966. doi: 10.1038/s41598-021-01513-x (PMC8578551; doi:10.1038/s41598-021-01513-x)
Supplement: Supplementary file 1 — Supplementary Information. [file 41598_2021_1513_MOESM1_ESM.docx]

**Supporting Information**

**3-D Vascularized Breast Cancer Model to Study the Role of Osteoblast in Formation of a Pre-Metastatic Niche**

*Rahul Rimal, Prachi Desai, Andrea Bonnin Marquez, Karina Sieg, Yvonne Marquardt, Smriti Singh^*^*

R. Rimal, P. Desai, A. B. Marquez, K. Sieg, Dr. S. Singh

DWI - Leibniz Institute for Interactive Materials, Forkenbeckstrasse 50, 52074 Aachen, Germany

Y. Marquardt

Department of Dermatology and Allergology, University Hospital, RWTH Aachen University, 52074 Aachen, Germany

Dr. S. Singh

Max Planck Institute for Medical Research, Jahnstrasse 29, 69120 Heidelberg, Germany

[smriti.singh@mr.mpg.de](mailto:smriti.singh@mr.mpg.de)


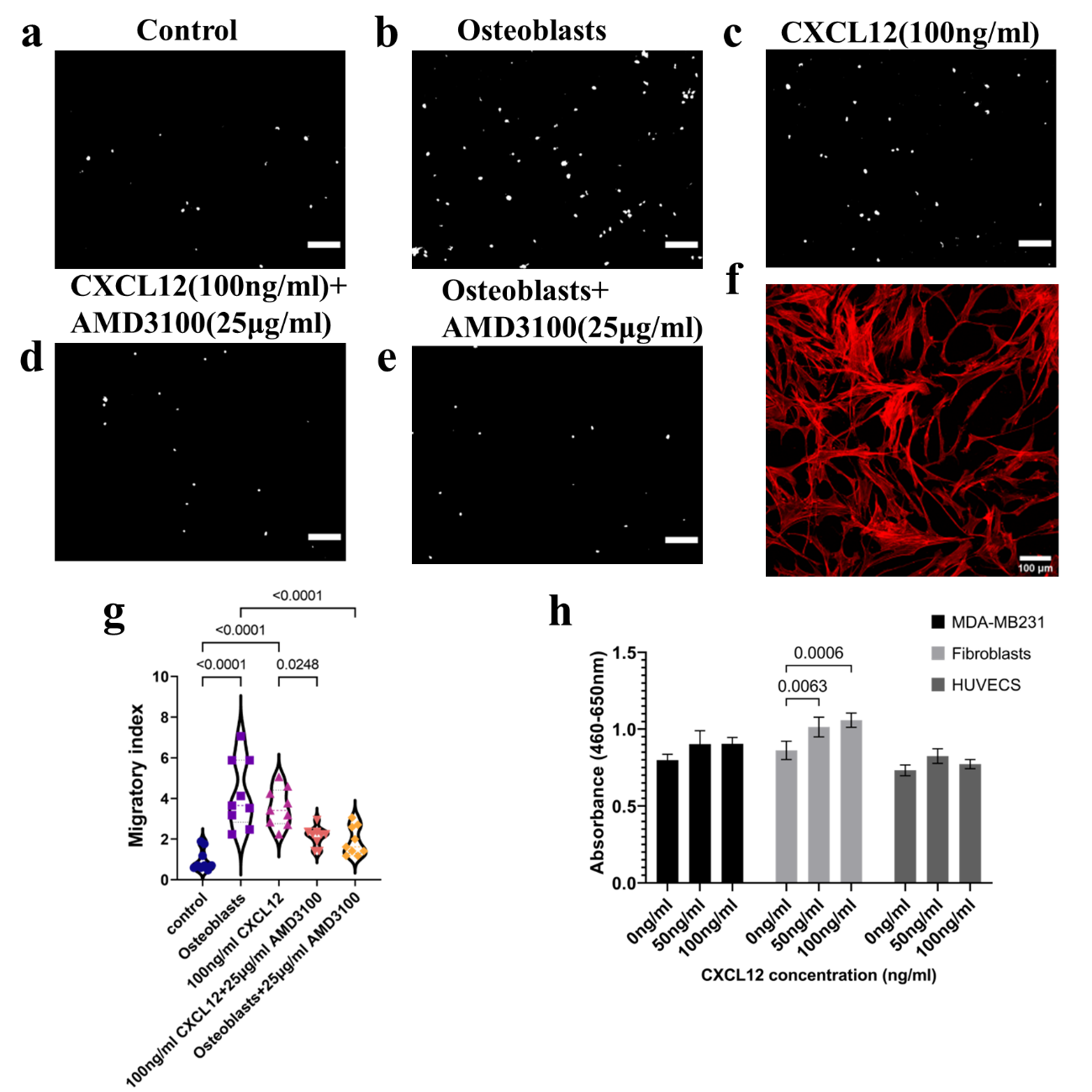


**Figure S1.** Migration of MDA-MB231 in a) Serum free media (control), b) Presence of osteoblasts in the well-plate in serum-free media c) Presence of 100 ng/ml of CXCL12 at the bottom of the insert in serum-free media d) Pre-treated MDA-MB231 with 25 µg/ml AMD3100 slower migration towards CXCL12 e) Pre-treated MDA-MB231 with 25 µg/ml AMD3100 slower migration towards osteoblasts, scale bar=100 μm f) morphology of OBs on the well plate, scale bar=100 μm g) significant difference in migration of MDA-MB231 in media vs osteoblasts and media vs CXCL12 added in the bottom, significant difference in migration observed after pre-treatment with AMD3100 towards osteoblasts and CXCL12 (n=3, with 3 different regions of the image) h) XTT assay shows slight increase in absorbance values in BCCs and HUVECs treated with 50 ng/ml and 100 ng/ml of CXCL12 and significant increase in Fibroblasts treated with 50 ng/ml and 100 ng/ml CXCL12 (n=3). Statistics used was ordinary one-way Anova with Tukey test.


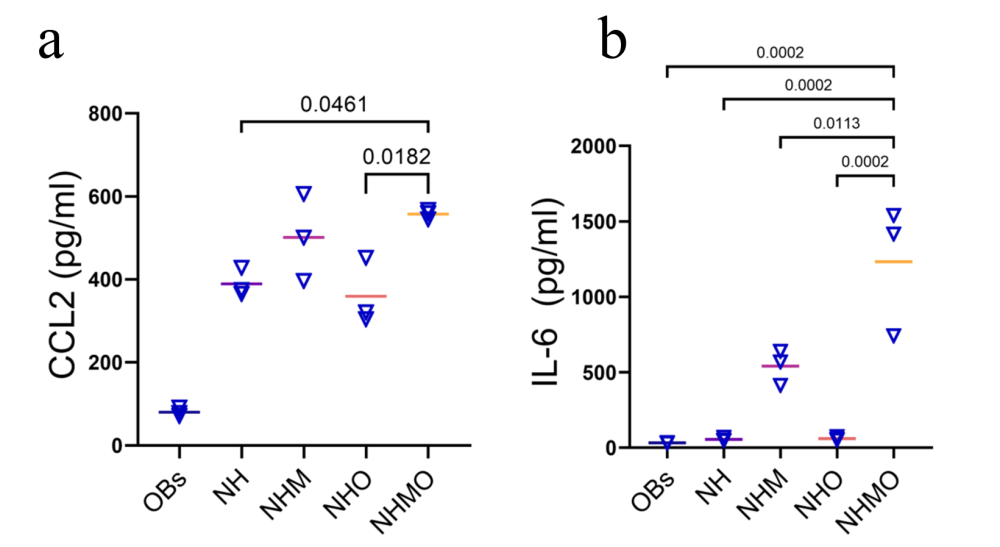


**Figure S2 Anaylsis of CCL2 and IL-6 a)** CCL2 analysis shows a significant difference in CCL-2 levels between NH and NHMO, NHO and NHMO, (n=3) b) Analysis of IL-6 levels by ELISA show significant difference in IL-6 detection between only OBs and NHMO, NH and NHMO, NHM and NHMO, NHO and NHMO. Values in the graphs are mean ± standard deviation; analysis was done using ordinary one-way Anova.


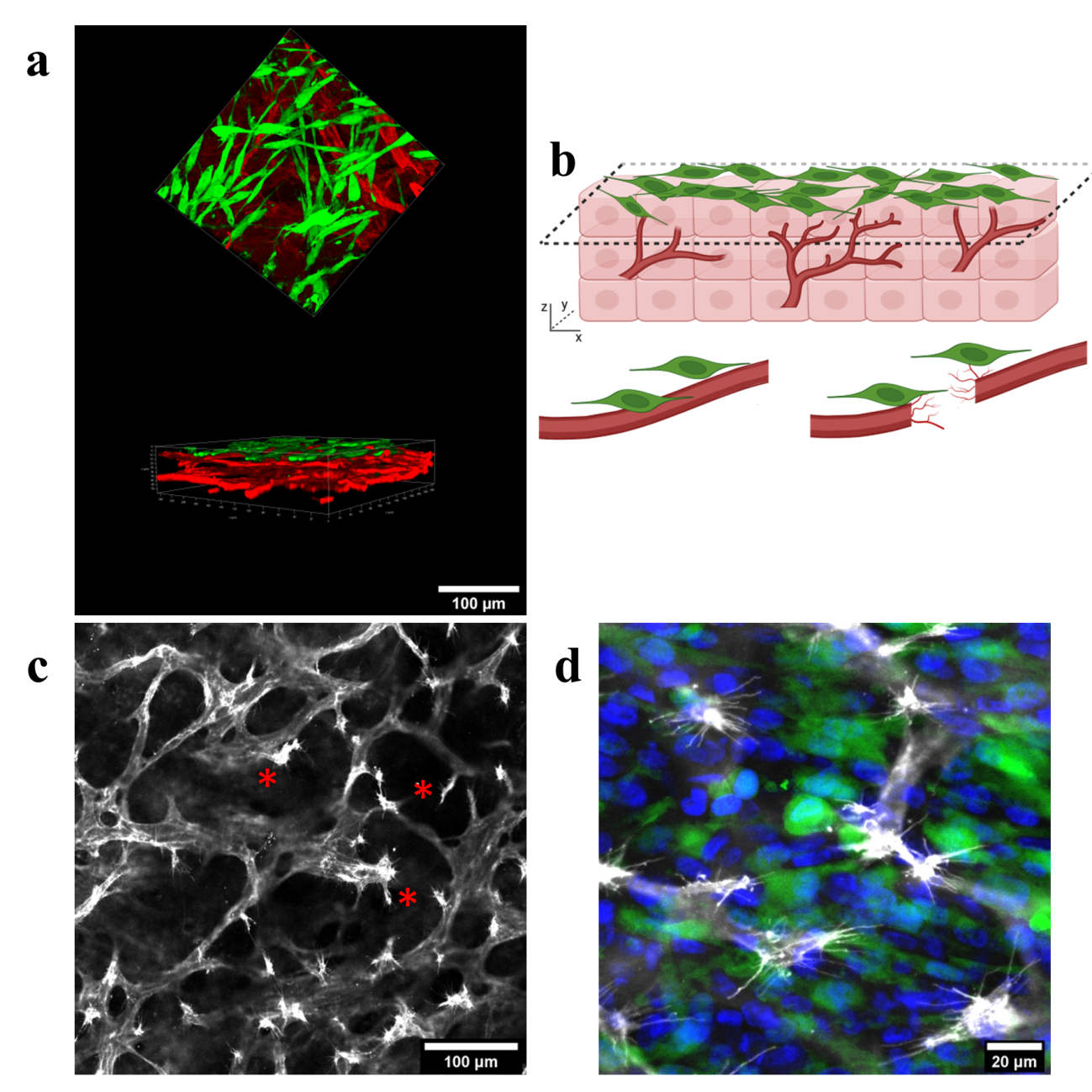


**Figure S3** a) Top view and side view of MDA-MB231(green) seeded on top of vascularized tissue (BVs= PECAM-1 stained red), scale=100 µm b) schematic illustration of the fate of vessels in direct contact with BCCs c) confocal images show broken vascular endpoints when in contact with MDA-MB231 cells, scale=100 µm d) magnified image shows the endpoints with sprouts (vessels= PECAM-1 stained, false colored gray, MDA-MB231=green), scale bar=20 µm


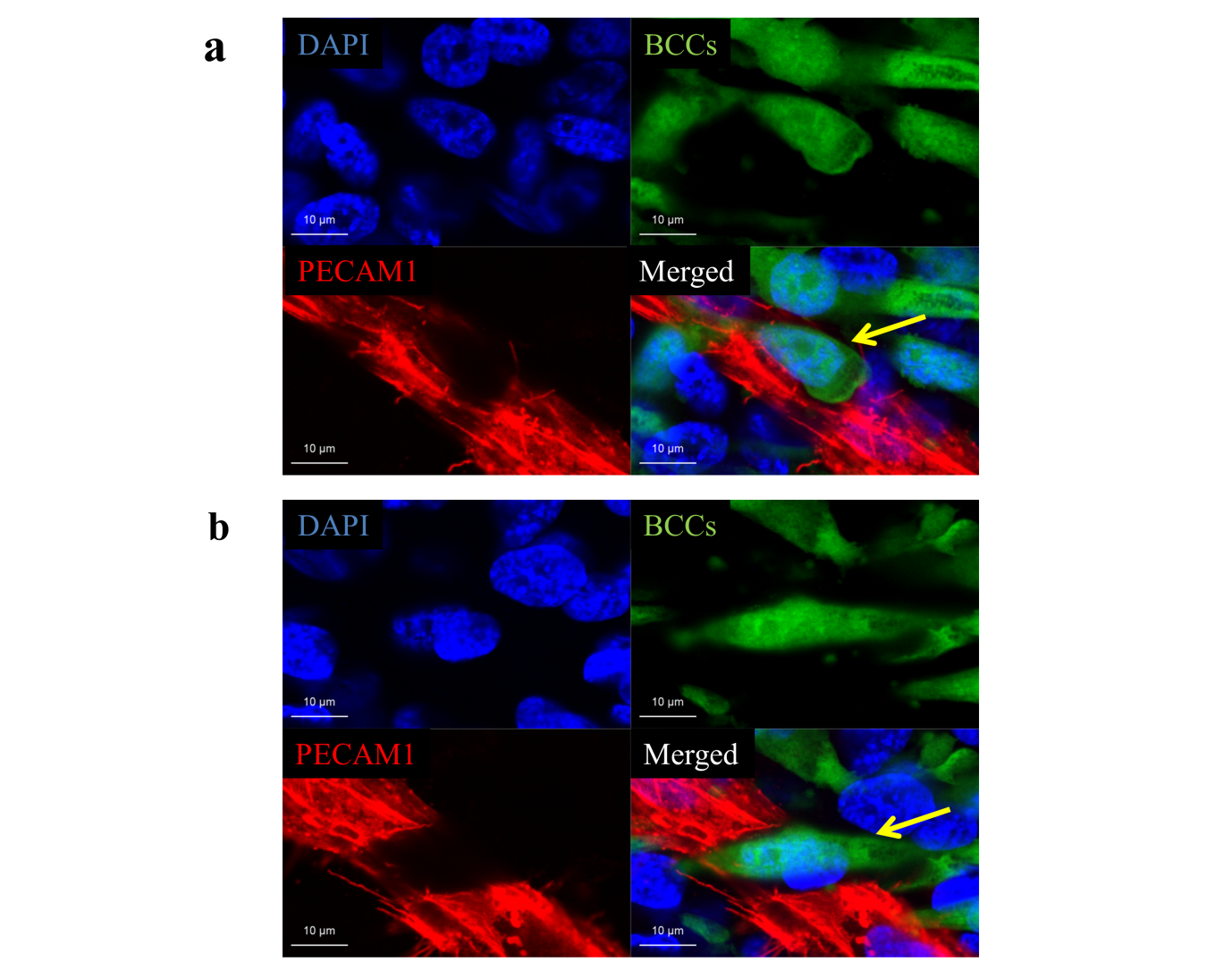


**Figure S4** a) Direct cell-cell contact between BCCs and vessels (DAPI=blue, BCCs=green, PECAM-1=red), scale bar=10μm b) BCCs penetrates and sections the vessels during direct contact, scale bar=10 μm

**Table S1 shows the expression values of the upregulated hub genes (NHMO vs NHM)**

| **Gene symbol** | **Gene name** | **Expression** | **FC** | **P-value** |
| --- | --- | --- | --- | --- |
| ***CYCS*** | Cytochrome C, Somatic | **up** | **2.03** | 0.0001 |
| ***EGFR*** | Epidermal Growth Factor Receptor | **up** | **2.28** | 3.12E-05 |
| ***HSP90N*** | heat shock protein 90 | **up** | **2.17** | 1.60E-03 |
| ***RPS27A*** | ribosomale 40S-Protein S27a | **up** | **2.09** | 1.63E-06 |


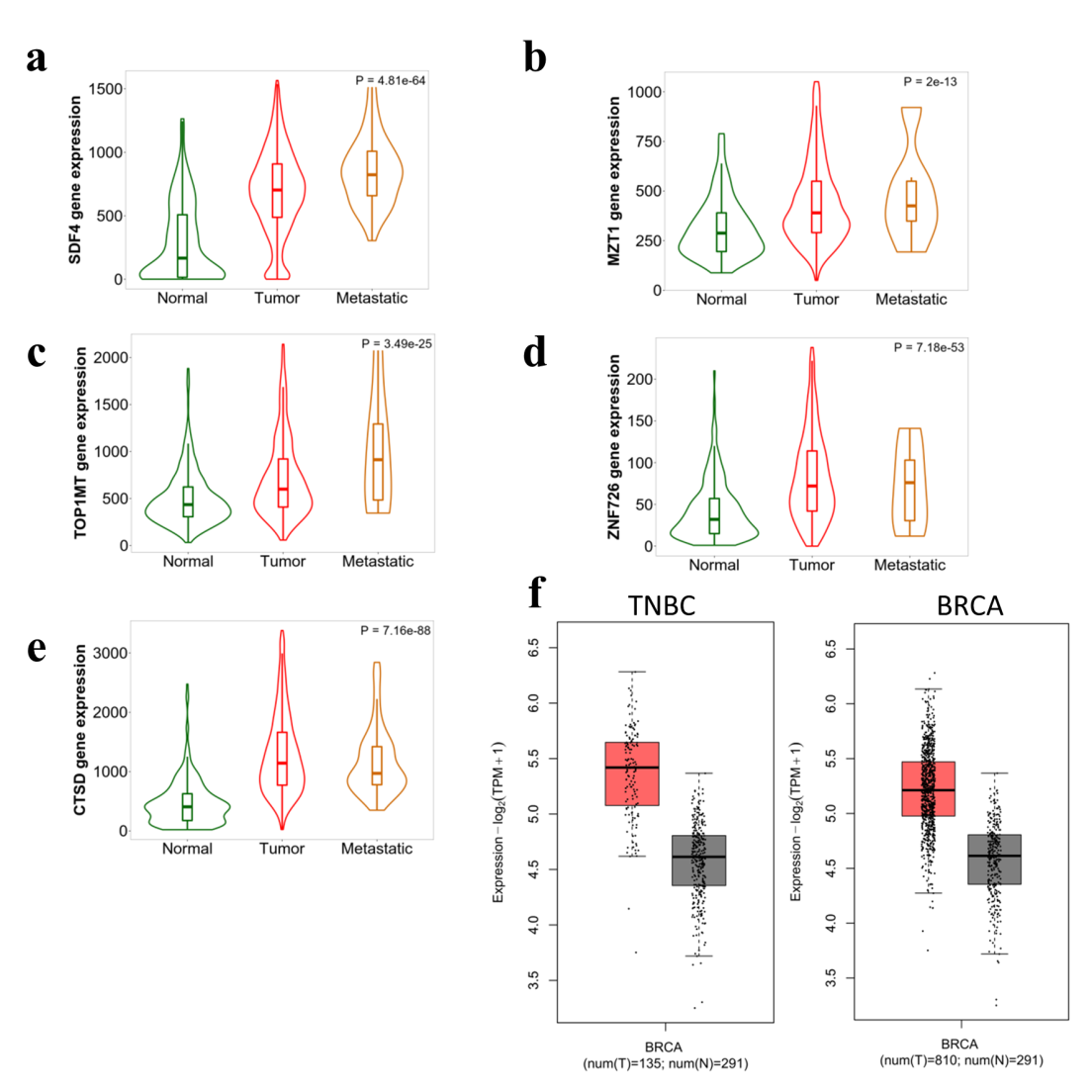


**Figure S54** Expression of the top five upregulated genes (NHMO vs NHM) in normal, tumor and metastatic tissue patient database (TNM plotter). Expression analysis of a) SDF4, b) MZT1, c) TOP1MT, d) ZNF726, and e) CTSD show higher expression of the genes in metastatic BRCA tissues as compared to normal breast tissues. f) The gene signature comprising of the top five upregulated genes were fed to the GEPIA2 online software. The gene signature is observed to be expressed more in specific TNBC tissue and overall BRCA tissue as compared to normal tissue.


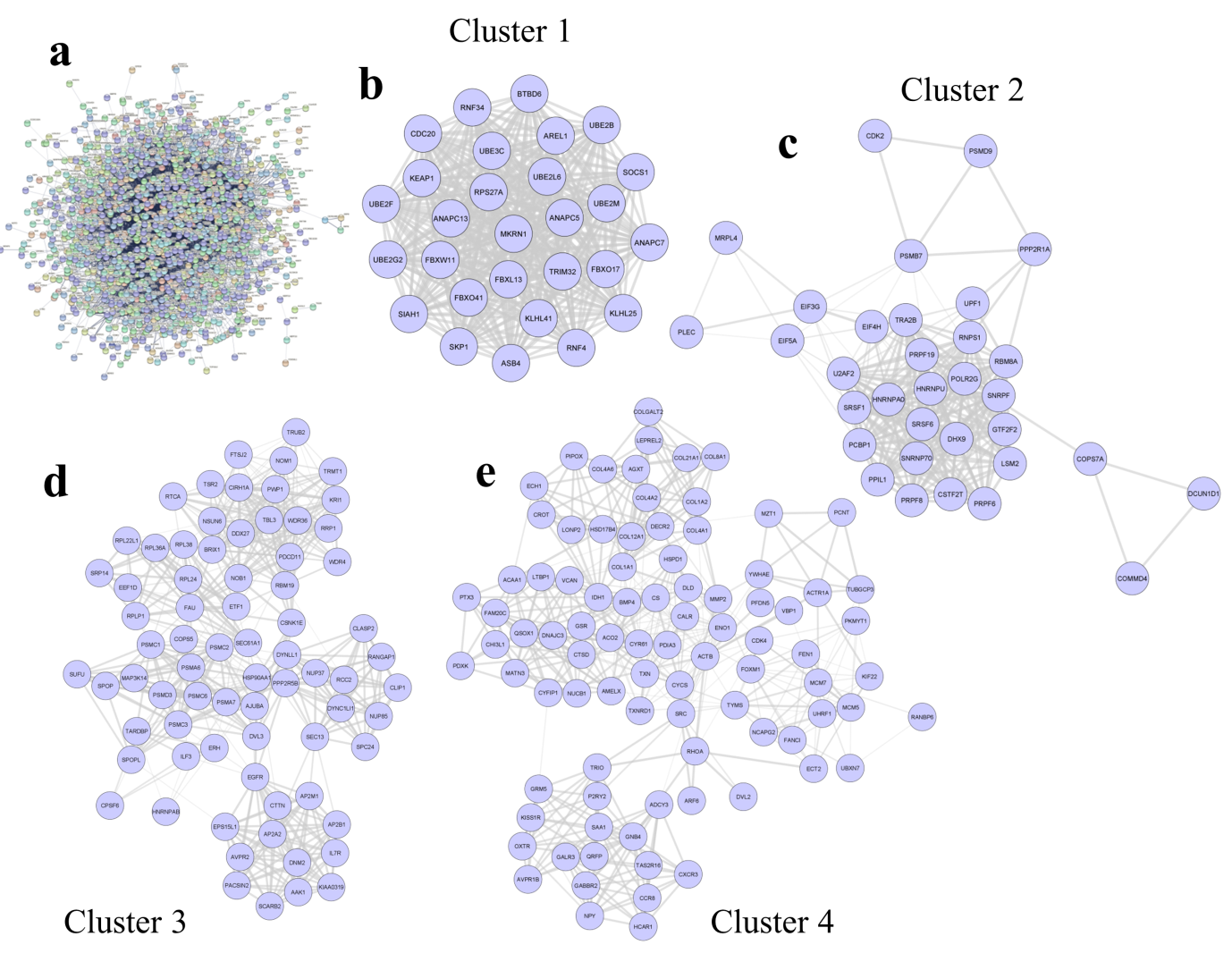


**Figure S6.** Upregulated protein clusters in NHMO vs NHM a) Overall upregulated protein-protein interaction network b, c, d, e) Four highly upregulated upregulated clusters (Clusters 1, 2, 3, and 4) acquired from MCODE application in the Cytoscape software.

**Table S2** shows the highly interconnected upregulated clusters (NHMO vs. NHM) and the genes involved in each cluster.

| **Cluster** | **Reactome Pathway** | **-Log(p)** | **Gene list** |
| --- | --- | --- | --- |
| ***Cluster 1*** | - Antigen Processing: Ubiquitination and Proteasome degradation | **47.54** | KEAP1, SKP1, UBE2M, MKRN1, ANAPC5, FBXW11, UBE2B, RPS27A, KLHL41, UBE2L6, FBXO17, UBE3C, ASB4, FBXL13, SOCS1, KLHL25, UBE2G2, AREL1, SIAH1, CDC20, RNF34, BTBD6, ANAPC7, TRIM32, ANAPC13, RNF4, FBXO41, UBE2F |
|  | - Neddylation | **15.60** | KEAP1, SKP1, UBE2M, FBXW11, RPS27A, KLHL41, FBXO41, ASB4, FBXL13, KLHL25, BTBD6, FBXO41, UBE2F |
| ***Cluster 2*** | - mRNA splicing- Major pathway | **29.05** | PRPF19, SRSF6, SRSF1, PRPF6, SNRPF, HNRNPU, POLR2G, PCBP1, U2AF2, HNRNPA0, CSTF2T, GTF2F2, DHX9, PPIL1, LSM2, TRA2B, RNPS1, PRPF8, RBM8A, SNRNP70 |
|  | - Metabolism of RNA | **25.83** | PRPF19, SRSF6, SRSF1, PSMB7, PRPF6, SNRPF, HNRNPU, POLR2G, PCBP1, U2AF2, HNRNPA0, PPP2R1A, CSTF2T, GTF2F2, DHX9, PPIL1, LSM2, TRA2B, PSMD9, RNPS1, PRPF8, RBM8A, UPF1, SNRNP70 |
| ***Cluster 3*** | - Separation of sister chromatids | **17.10** | PPP2R5B, NUP85, PSMC1, PSMA6, PSMD3, DYNC1LI1, PSMA7, RCC2, SEC13, DYNLL1, PSMC2, RANGAP1, PSMC6, CLASP2, NUP37, SPC24, CLIP1, PSMC3 |
|  | - Metabolism of RNA | **17.06** | FTSJ2, NUP85, PSMC1, PSMA6, PSMD3, NOB1, RPL38, CIRH1A, RPLP1, RPL22L1, ETF1, PDCD11, PSMA7, NSUN6, RPL24, CSNK1E, WDR4, PSMC2, PSMC6, RPL36A, RRP1, WDR36, NUP37, TBL3, TRMT1, PSMC3 |
| ***Cluster 4*** | - Protein localization | **9.42** | ACO2, DECR2, ECH1, LONP2, AGXT, PIPOX, ACAA1, CS, HSPD1, IDH1, CROT, HSD17B4 |
|  | - Collagen biosynthesis and modifying enzymes | 9.42 | COL1A1, COL21A1, COL8A1, COL1A2, COL12A1, COL4A2, COLGALT2, COL4A1, COL4A6, LEPREL2 |

**
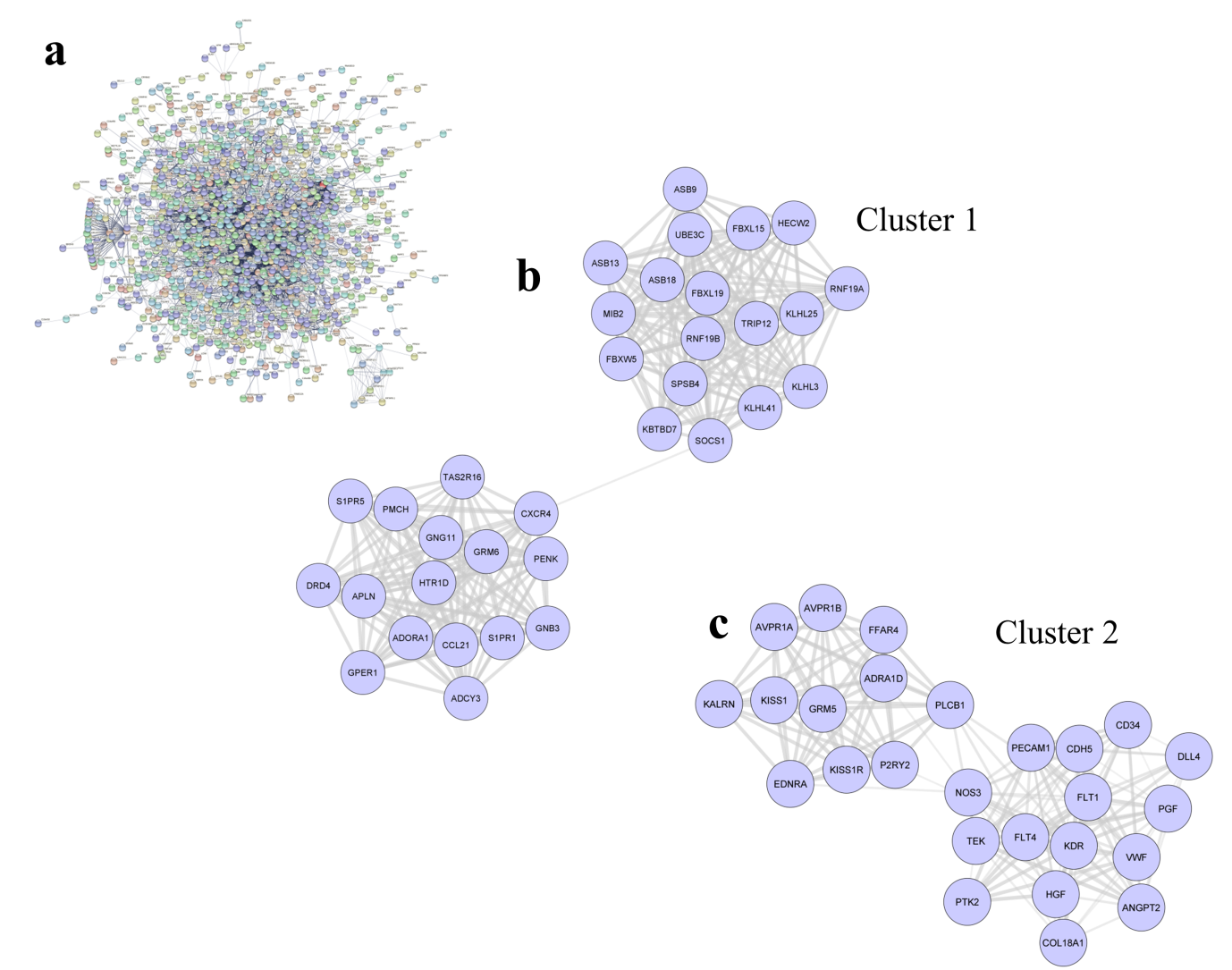
**

**Figure S7.** Upregulated protein clusters in NHMO vs NMO a) Overall upregulated Protein-protein interaction network, b, c) Two highly interconnected upregulated clusters (clusters 1 and 2) acquired from MCODE application in the Cytoscape software.

**Table S3** Upregulated gene ontology and gene symbols (NHMO vs NMO)

| **Category** | **Term** | **-Log(p)** | **Gene symbols** |
| --- | --- | --- | --- |
| GO:0030855 | - Epithelial cell differentiation | 7.702 | BMP2,CD34,CDH5,CDSN,COL4A1,CYP1A1,DAB2,DLX3,S1PR1,EVPL,FLNA,NR5A2,B4GALT1,ID1,ID2,ID3,IL1B,JAG2,JUP,KDR,KRT5,KRT9,KRT15,KRT86,LORICRIN,MEF2C,AFDN,PECAM1,PODXL,POU4F3,PRKCH,PTK6,S100A7,SOX4,SPRR2G,SPRR3,ST14,TBX1,TCF15,TIE1,TJP1,CXCR4,PKP4,KRT75,TGM5,SLC4A7,RAPGEF2,MTSS1,CCNO,KLF2,UPK1A,PLCB1,HEY1,SCRIB,MAFF,PPP1R16B,ABCA12,LCE2B,F11R,SOX18,COL18A1,KRTAP4-5,IL31RA,KRT72,KRTAP15-1,KRTAP6-1,KRTAP20-1,KRT79,LIPM,LCE1C,LCE1D,KRTAP12-1,KRTAP10-6,KRTAP10-5,KRTAP5-4,MIR10A,KRTAP5-7,CCDC88C,LIPN,COL1A2,COMP,INHBA,ITGA6,SRF, AP3B1,HLA-DRB1,,KRTAP21-1, KRTAP29-1 |
| GO:0006935 | - Chemotaxis | 5.295 | ADAM8,ANGPT2,CDH13,LYST,CXADR,S1PR1,EFNA1,EGR2,EPHA3,EPHA4,EPHB1,FOXG1,FLT1,HGF,IL6R,KDR,KIF5A,LAMA2,MST1,NCAM1,NOTCH3,OR1D2,PDE4B,PGF,PIK3CB,PIK3CG,POU4F3,PPIB,PTK2,S100A7,CCL14,CCL15,CCL21,SEMA3F,SLC12A2,SPN,CXCR4,SEMA3B,PLA2G10,KALRN,DOCK4,SEMA6B,SEMA4B,DPYSL4,B3GNT2,PLXND1,SCRIB,DAPK2,PRKD2,ARHGEF16,LEF1,SEMA5B,LTB4R2,DPYSL5,PLEKHG5,MTUS1,PREX1,MMP28,SHANK3,TIRAP,EMB,CMTM2,UNC5B,DEFB109B,ECSCR,FLNA,ID1,TACSTD2,MEF2C,MYH9,NPTX1,SRF,ST14,CDKL5,TBCD,TEK,USP9X,FZD4,RAPGEF2,KIAA0319,PLPPR4,WDR1,NBEAL2,COBL,PPP1R9A,RCC2,HECW2,TNN,COL18A1,FERMT3,ACTBL2,CCDC88C,SYT1,UGT8,RASAL1,RIMS1,SYT17,CPNE5,CACNG7,NES,ABLIM1,RGS3,RNASE1,ABLIM2,MYL12B,FKBP1B |
| GO:0030335 | - Positive regulation of cell migration | 5.169 | ADAM8,BMP2,CDH5,CDH13,DAB2,S1PR1,ETS1,FLNA,FLT1,FLT4,GPER1,HGF,HYAL1,IL1B,IL6R,ITGA6,ITGA2B,KDR,MCAM,MMP14,NOS3,PECAM1,PGF,PIK3CB,PIK3CG,PODXL,PTK2,S100A7,CCL21,SEMA3F,SPN,TEK,TJP1,CXCR4,SEMA3B,RAPGEF2,DOCK4,HDAC9,SEMA6B,SEMA4B,PLK2,CLASP2,DAPK2,SUN2,PRKD2,NOX4,LEF1,SEMA5B,PREX1,DOCK8,PLVAP,CD99L2,FERMT3,TIRAP,MIR10A,SMIM22,FAM110C,MIR939,RNASE10,CHMP4C,ANGPT2,MST1,MTUS1,MMP28 |
| hsa05200 | - Pathways in cancer | 5.009 | ADCY3,AREG,BAK1,BMP2,CASP7,CCND3,COL4A1,CSF2RB,E2F1,EDNRA,EPAS1,EPOR,ETS1,MECOM,FGF11,FLT4,GNAQ,GNB3,GNG11,MSH6,HGF,IFNA2,IFNA5,IFNAR1,IL2RA,IL3RA,IL6R,IL7,IL12B,ITGA6,ITGA2B,JAG2,JUP,LAMA2,MMP1,NOTCH3,PGF,PIK3CB,PMAIP1,PTK2,RAD51,RNASE1,RXRA,TGFB3,ZBTB17,CXCR4,FZD4,TNFRSF25,LONP1,PIM2,PLCB1,HEY1,DAPK2,RASGRP3,LEF1,DLL4,PLEKHG5,EGLN3,IL23R,BMP3,TNFRSF8,FLT1,GDF10,IL1B,IL1RAP,INHBA,KDR,CCL14,CCL15,CCL21,TNFSF4,TNFSF10,IL32,IL20RB,CRLF2,IL25,RELT,IL31RA,CCL15-CCL14,IL31,SOCS1 |

**Table S4** Downregulated gene ontology terms and gene symbols (NHMO vs. NMO)

| **Category** | **Term** | **-Log(p)** | **Gene symbols** |
| --- | --- | --- | --- |
| GO:1905114 | - Cell surface receptor signaling pathway involved in cell-cell signaling | 6.65 | ADRB2,XIAP,BMP4,C3,CCNE1,CSNK1G3,CELSR3,PTK2B,GPC3,GLRB,IGFBP6,RBPJ,ISL1,LRP6,SALL1,TLE1,TRPV1,AXIN2,RECK,TNKS,DKK1,KANK1,GREM1,AMOTL2,WNT4,VPS35,KREMEN1,NKD2,STX1B,APCDD1,TMEM64,AGO4,TMEM9,RSPO1,H2BC5,H2AC4,SOX6,H4-16,ITPR3,MIR16-1 |
| GO:0048608 | - Reproductive structure development | 5.34 | BMP4,C3,CBL,ERCC1,FOXF2,HOXA13,RBPJ,ITGB8,LRP6,PGR,PTN,PTX3,SALL1,SLIT3,ZP3,STC2,ADAMTS1,FST,RHOBTB3,WNT4,ETNK2,LHX4,AGO4,MIR16-1,DACH1,PTK2B,NME5,NANOS1,MEIG1,PRDM9,AANAT,ADCY1,ASS1,PTGDS,TPH1,THRAP3,NMU |
| GO:0030278 | - Regulation of ossification | 5.12 | ADRB2,BMP4,PTK2B,RBPJ,LRP6,MGP,PTN,TPH1,AXIN2,SEMA4D,DKK1,GREM1,WNT4,KREMEN1,OSR2,TMEM64,GPC3,MN1,RPS15,FAT4,CHRDL1,IGSF10,FAM83H,CRB1,MIR16-1 |
| GO:0003128 | - Heart field specification | 4.74 | BMP4,RBPJ,ISL1,AXIN2,DKK1,LRP6,PTN,SALL1,HAP1,WNT4,ASS1,CASQ1,CENPF,CRYAB,FGF2,G6PD,HLX,MYL3,MYL6,SMTN,SVIL,SAP30,GREM1,SOX6,EID2B,MKX,CXCL14,BOC,GPC3,HOXA13,PCSK5,SLIT3,ADAMTS1,HSPB7,TMEM100,FAT4,XIRP2,CCN3,FOXF2 |
| GO:0071772 | - Response to BMP | 4.34 | XIAP,BMP4,GDF1,GPC3,HOXA13,RBPJ,TFAP2B,UBE2D3,FST,GREM1,TMEM100,CHRDL1,VSTM2A,ANGPT1,APOB,C3,EPHB3,PTK2B,FGF2,ISL1,ITGB8,CCN3,PTN,SPI1,TNFAIP2,RECK,ANGPTL1,ADAMTS1,CXCL13,SPINK5,TNFRSF12A,AMOTL2,WNT4,SMOC2,HSPB6,MIR15A,NTF3,CBL,CILP,DKK1,ZC3H3,GALNT3,HAP1,SOX6,FNDC4,FAT4,MIR16-1 |
| GO:0035766 | - Cell chemotaxis to fibroblast growth factor | 4.28 | FGF2,CXCL13,MIR15A,CCN3,SMOC2,CBL,GALNT3,WNT4,FAT4, MIR16-1 |
| GO:0044057 | - Regulation of system process | 4.27 | ADRB2,CACNB2,CACNB4,CASQ1,CHRM2,PTK2B,G6PD,GPR35,GSTM2,ISL1,ITPR3,KCND3,MAG,MYL3,CCN3,SCN4A,SCN4B,SLC25A12,DLGAP1,NMU,CACNG5,HSPB7,EHD3,TMEM100,RAB11FIP1,TRIM63,STX1B,HSPB6,CRYAB,MYL6,SMTN,SSPN,SORBS1 |
| GO:0055080 | - Cation homeostasis | 4.25 | XIAP,CACNB2,CASQ1,PTK2B,FGF2,GPR35,GRINA,GSTM2,ITPR3,LRP6,SLC26A4,CCL13,CXCL11,TFAP2B,TRPV1,STC2,HAP1,CXCL13,NMU,SLC7A8,LPAR3,ACKR4,STEAP4,KCTD17,COX19,SLC9B1,TMTC2,SLC30A8,TMEM64,TMEM9,MICU3,SCARA5,ADRB2,RGS9,EHD3,LRRC26,C3,G6PD,GPC3,IRS2,SORBS1,CACNB4,CACNG5,IL16,HOMER3,PKD1L2,KCND3,SCN4A,SCN4B,CLIC3,STXBP4,HCN1,COX8A,SLC15A2,ATP5F1EP2,FABP3 |
